# Lithium monitoring in patients over 65 in NHS Greater Glasgow and Clyde

**DOI:** 10.1192/bjo.2021.138

**Published:** 2021-06-18

**Authors:** Catriona Ingram, Karli Dempsey, Gillian Scott, Joe Sharkey

**Affiliations:** 1 NHS Greater Glasgow and Clyde; 2 NHS Lanarkshire; 3 NHS Ayrshire and Arran

## Abstract

**Aims:**

Our aim was to identify current practice for Lithium monitoring for >65s in NHS GGC and assess compliance to local Lithium monitoring guidelines.

**Method:**

A retrospective analysis was undertaken of patient data (demographics, diagnosis, biochemistry results) with Caldicott approval at two points over the course of 2018/19. For the first analysis, old age Community Mental Health Teams (CMHTs) were approached and asked to provide a list of their patients on Lithium. This was then assessed for compliance to Lithium monitoring guidelines.

For the second analysis, pharmacy provided data for every patient in the health board dispensed lithium, regardless of whether they were open to a CMHT or not. We were then able to identify patients who we had not picked up on our initial analysis, and re-assess the entire data set for compliance to Lithium monitoring guidelines.

**Result:**

From our first analysis, 13 CMHTs identified 155 patients on Lithium. There was a high variability in how these patients were identified. 44% of patients were monitored by CMHTs who took bloods and chased them, 38% were monitored by GPs who were prompted by CMHTs in routine clinic letters, and 14% were monitored by GPs who were prompted by CMHTs more assertively using a lithium register. Overall, Lithium plasma monitoring was done well irrespective of method (91%), however compliance to the local standards was poor (58%) with proactive CMHT prompting GPs appearing to be the most effective method (71%).

In our second analysis, we identified 508 patients >65 in NHS GGC prescribed Lithium. Of those, 44% were open to old age psychiatry, 25% general adult psychiatry and 19% were not open to anyone. Of those open to old age services, only 58% had been identified in the previous audit. Lithium monitoring compliance was better in those open to a CMHT versus those not (61% to 23%), and better in CMHTs where monitoring was done by CMHTs rather than GPs. For each CMHT, there were roughly 7 patients per catchment area on Lithium not open to psychiatry.

**Conclusion:**

Lithium monitoring does appear to be highly variable and not particularly compliant with local standards. CMHTs have inconsistent methods of identifying patients prescribed Lithium. There are a significant number of patients not open to old age CMHTs prescribed Lithium, and these patients have poorer compliance to Lithium monitoring. Of patients open to CMHTs, CMHT-led monitoring appears superior to other forms.

